# Supplementary material for: A Study of a Protein-Folding Machine: Transient Rotation of the Polypeptide Backbone Facilitates Rapid Folding of Protein Domains in All-Atom Molecular Dynamics Simulations
Source: Int J Mol Sci. 2023 Jun 13;24(12):10049. doi: 10.3390/ijms241210049 (PMC10298387; doi:10.3390/ijms241210049)
Supplement: Supplementary file 1 [file ijms-24-10049-s001.zip › Figure S1.pdf]

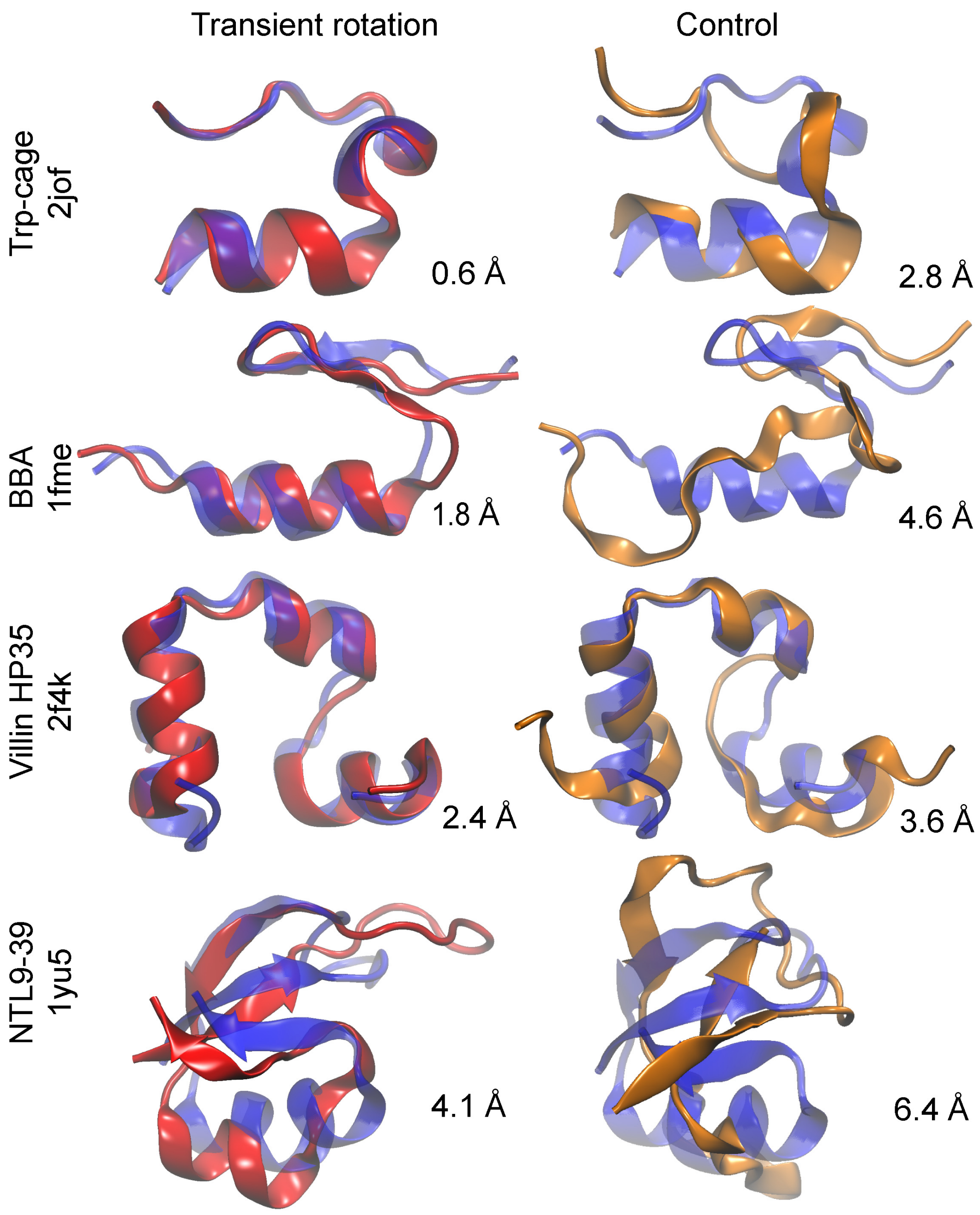

Transient rotation
Control
Trp-cage
2jof
0.6 Å
2.8 Å
BBA
1fme
1.8 Å
4.6 Å
Villin HP35
2f4k
2.4 Å
3.6 Å
NTL9-39
1yu5
4.1 Å
6.4 Å

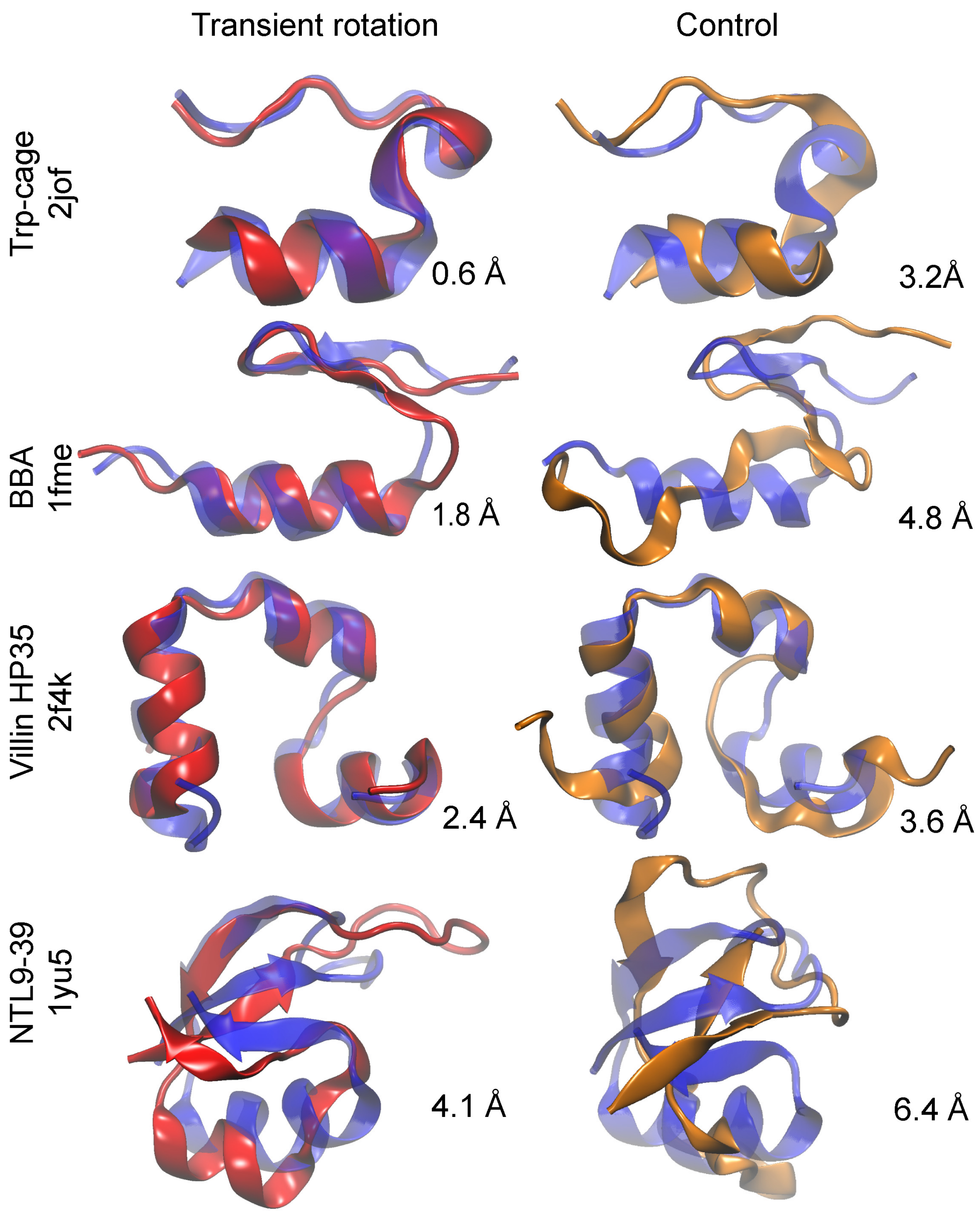

Figure S1. The snapshots from MD simulations with transient rotation (red) or unassisted controls (orange) superimposed onto the known three-dimensional structure of the targets (blue). In the simulations with transient rotation, the frame with the smallest RMSD distance to the known structure among all three trajectories was selected. In the first set of figures, for the control simulations the form with the smallest RMSD distance to the known structure among all three trajectories was selected as well. In the second set of figures, the frames for the rotation-assisted and control simulations were taken at the same time point, i.e., when the rotation-assisted simulation adopted the structure with the smallest RMSD distance to the known structure. The RMSD distance in angstrom is shown beside each superimposition.
