# Supplementary material for: A Study of a Protein-Folding Machine: Transient Rotation of the Polypeptide Backbone Facilitates Rapid Folding of Protein Domains in All-Atom Molecular Dynamics Simulations
Source: Int J Mol Sci. 2023 Jun 13;24(12):10049. doi: 10.3390/ijms241210049 (PMC10298387; doi:10.3390/ijms241210049)
Supplement: Supplementary file 1 [file ijms-24-10049-s001.zip › Table S1.pdf]

Table S1. Properties of simulation boxes used in this study

Protein domain	Simulation type	X	Y	Z	# atom	# K/Cl	# water
Trp-cage	Rotation	7.81	4.33	4.33	14885	9/9	4861
	Control *	7.81	4.33	4.33	14885	9/9	4861
BBA	Rotation	10.89	5.31	5.31	31110	19/23	10188
	Control	6.02	6.02	6.02	15391	9/13	4955
Villin	Rotation	9.82	4.29	4.29	17805	11/13	5734
	Control	9.82	4.29	4.29	17805	11/13	5734
NTL9	Rotation	13.09	4.03	4.03	20931	13/16	6760
	Control	13.09	4.03	4.03	20931	13/16	6760

* one of the control runs was performed in the cubic box with sides 4.54x4.54x4.54
